# Ensemble of structure and ligand-based classification models for hERG liability profiling

**DOI:** 10.3389/fphar.2023.1148670

**Published:** 2023-03-23

**Authors:** Serena Vittorio, Filippo Lunghini, Alessandro Pedretti, Giulio Vistoli, Andrea R. Beccari

**Affiliations:** ^1^ Dipartimento di Scienze Farmaceutiche, Università Degli Studi di Milano, Milano, Italy; ^2^ EXSCALATE, Dompé Farmaceutici SpA, Napoli, Italy

**Keywords:** hERG, cardiotoxicity, predictive toxicology, machine learning, random forest, docking

## Abstract

Drug-induced cardiotoxicity represents one of the most critical safety concerns in the early stages of drug development. The blockade of the human ether-à-go-go-related potassium channel (hERG) is the most frequent cause of cardiotoxicity, as it is associated to long QT syndrome which can lead to fatal arrhythmias. Therefore, assessing hERG liability of new drugs candidates is crucial to avoid undesired cardiotoxic effects. In this scenario, computational approaches have emerged as useful tools for the development of predictive models able to identify potential hERG blockers. In the last years, several efforts have been addressed to generate ligand-based (LB) models due to the lack of experimental structural information about hERG channel. However, these methods rely on the structural features of the molecules used to generate the model and often fail in correctly predicting new chemical scaffolds. Recently, the 3D structure of hERG channel has been experimentally solved enabling the use of structure-based (SB) strategies which may overcome the limitations of the LB approaches. In this study, we compared the performances achieved by both LB and SB classifiers for hERG-related cardiotoxicity developed by using Random Forest algorithm and employing a training set containing 12789 hERG binders. The SB models were trained on a set of scoring functions computed by docking and rescoring calculations, while the LB classifiers were built on a set of physicochemical descriptors and fingerprints. Furthermore, models combining the LB and SB features were developed as well. All the generated models were internally validated by ten-fold cross-validation on the TS and further verified on an external test set. The former revealed that the best performance was achieved by the LB model, while the model combining the LB and the SB attributes displayed the best results when applied on the external test set highlighting the usefulness of the integration of LB and SB features in correctly predicting unseen molecules. Overall, our predictive models showed satisfactory performances providing new useful tools to filter out potential cardiotoxic drug candidates in the early phase of drug discovery.

## 1 Introduction

Toxicity is the main cause of drugs failures during all the stages of drug development as well as of their withdrawal from the market (Kramer et al., 2007; Lunghini et al., 2022). Several studies highlighted that cardiotoxicity and hepatotoxicity represent the most frequent adverse effects registered during clinical trials and post-approval surveillance (Schuster et al., 2005; Guengerich, 2011). Drug-induced cardiotoxicity is often related to the off-target inhibition of the human ether-a-go-go-related gene (hERG) potassium channel, a voltage-gated potassium channel involved in the repolarization of the cardiac action potential (Garrido et al., 2020). The blockade of hERG by drugs might lead to the increment of the duration of the cardiac action potential, a condition noted as QT prolongation, that could result in fatal arrythmias (Vandenberg et al., 2012). For this reason several drugs like astemizole (Zhou et al., 1999), terfenadine (Tanaka et al., 2014), cisapride (Mohammad et al., 1997), brompheniramine (Park et al., 2008) and chlorpromazine (Thomas et al., 2003) have been withdrawn from the market. Therefore, assessing hERG liability of new drug candidates in the early stages of the drug discovery process is important to prevent undesired cardiotoxic effects. For this purpose, several experimental methods based on electrophysiological techniques, fluorescence binding and atomic absorption have been developed to date (Danker and Möller, 2014). However, these assays are time-consuming, expensive, and therefore not suitable for the screening of large libraries. In this scenario, *in silico* approaches have emerged as attractive tools for the development of predictive models for hERG-related cardiotoxicity assessment being less costly and faster (Zhang et al., 2022). In recent years several ligand-based (LB) approaches, spanning from pharmacophore modelling to machine learning (ML) methods, have been exploited to generate computational models able to correctly identify potential hERG blockers. The first pharmacophore model for hERG inhibitors was published in 2002 by Ekins et al. (2002) and was composed by four hydrophobic and one positive ionizable features. In the same year, Cavalli et al. (2002) reported another pharmacophore model, along with a CoMFA study, which consisted of three aromatic ring connected by a nitrogen atom thus forming a tertiary amine group which is positively charged at physiological pH. Over the years, other ligand-based pharmacophore models were reported in literature (Aronov and Goldman, 2004; Aronov, 2006). However, these models are generated starting from relatively small training sets thus showing a limited applicability. Indeed, the capacity of hERG channel to bind different chemotypes has been widely recognized and this feature cannot be described by a common pharmacophore (Wang et al., 2016).

The advancement of ML algorithms in drug discovery programs promoted the development of predictive models for hERG liability, which are mainly based on physiochemical descriptors, fingerprints and graphs (Karim et al., 2021). Some recent examples include the study published by Zhang and co-workers who built different classification models based on 13 molecular descriptors and fingerprints by employing five ML methods. The model trained on the CDK fingerprints in combination with molecular descriptors by using the support vector machine (SVM) strategy achieved the best performance with an accuracy value of 0.8475 for the test set (Zhang et al., 2016). Lee et al. proposed CardPred which is a neural network model built on 3456 physicochemical descriptors and fingerprints which showed MCC values of 0.368 and 0.655 in ten-fold cross-validation and on an external test set, respectively (Lee et al., 2019). More recently, Zhang *et al.* developed HergSPred, a consensus model created by averaging the output of individual classifiers trained on Morgan and MACCS fingerprints by using three different ML methods: Random Forest, Deep Neural Network and Extreme Gradient Boosting. This consensus model reached an accuracy value of 0.840 on the test set 1 used in this study (Zhang et al., 2022). Apart from classification models used to discern between blockers and non-blockers, also QSAR studies aimed at predicting hERG binding affinity by regression analysis were performed. For instance, Du-Cuny et al. (2011) developed six *k*NN regression models with the best model obtained by using eight physicochemical descriptors having a *R* ^2^ value of 0.59. Arab and Barakat. (2022) have recently published a QSAR model based on 8380 compounds, by using Random Forest algorithm and employing 144 2D descriptors, obtaining a *R* ^2^ value of 0.67 on the test set.

Despite the good performances achieved, the main issue of the LB methods relies on the limited structural diversity covered by the data available in public repositories (Siramshetty et al., 2020). The reliability of a LB model strictly relies on the similarity of the studied molecules in respect to the training compounds and, therefore, they often fail to correctly predict unseen molecules characterized by new chemical scaffolds (Konda et al., 2019; Creanza et al., 2021).

Recently, the cryo-EM structure of hERG channel in complex with the inhibitor astemizole (PDB ID 7CN1) have been solved paving the way to the use of structure-based (SB) approaches, which are not dependent on the structural similarity, thus allowing to overcome the limitations of the LB methods.

**FIGURE 1:**
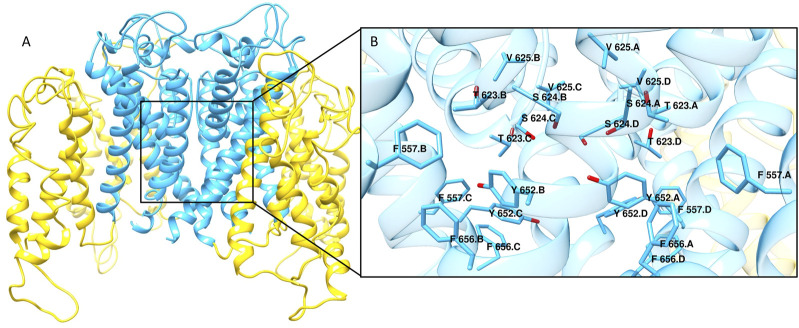
**(A)** Cryo-EM structure of hERG channel in complex with astemizole (PDB ID 7CN1). The α-helixes S1-S4 constituting the voltage sensor are shown as yellow ribbons, while cyan ribbons represent the core region of the channel. **(B)** Close view of the main ligand binding site for hERG blockers. The residues that are reported to be mainly involved in the interactions with hERG binders are displayed as cyan sticks.

hERG is a homotetrameric protein with each monomer composed of six transmembrane α-helixes. Specifically, helixes S1-S4 form the voltage sensor of the channel, while helixes S5 and S6 constitute the pore which contains the main binding site for drug-like molecules (Figure 1). In the 7CN1 structure, the voltage sensor is in the open conformation with the selectivity filter in the inactivated form (Asai et al., 2021). Due to the low quality of the EM density for astemizole, the inhibitor is not present in this structure. However, basing on the density position, the Authors proposed a possible binding mechanism for astemizole which is in good agreement with the mutagenesis data reported in literature according to which the mutations of residues Tyr652, Phe656, Thr623, Ser624, Val 625, Gly648 and Phe557 negatively affect the inhibitors affinity towards hERG channel (Chen et al., 2002; Perry et al., 2006; Dempsey et al., 2014; Saxena et al., 2016).

Among the SB approaches, one of the most used techniques in the drug discovery field is molecular docking, which allow to predict the binding conformation of a bioactive molecule to its biological target (Torres et al., 2019). Recent studies highlighted the use of molecular docking simulations for the development of reliable classification models for predictive toxicology (Trisciuzzi et al., 2017; Trisciuzzi et al., 2018). In this context, a successful application of docking methodology to hERG liability prediction was recently reported by Creanza *et al.*, who combined docking scores and ligand interaction fingerprints. The study involved the docking of 8337 known hERG binders to develop classification models by means of a support vector machine LASSO regularized approach. The best models reached accuracy values in the range of 0.76–0.79 when inactivity thresholds higher than 70 μM and the conformations derived from induced-fit docking experiments were used (Creanza et al., 2021). Instead, Meng *et al.* built a target specific scoring function, named TSSF-hERG, based on a training set of 9215 compounds. TSSF-hERG was based on interaction features, as calculated on the top-ranked conformations computed by AutoDock Vina, plus LB descriptors and was developed by applying support vector regression algorithms. The model showed a Pearson’s correlation coefficient (R) of 0.765 and a RMSE value of 0.585 in ten-fold cross-validation performed on the training set. TSSF-hERG outperformed the classical scoring function of AutoDock Vina and the generic scoring function RF-Score based on Random Forest algorithm (Meng et al., 2021). The key information about the above described models are summarized in Supplementary Table S1. Despite several predictive models for hERG-related cardiotoxicity based on both LB and SB attributes have been described in literature, to the best of our knowledge, a direct comparison between the two approaches has never been reported so far. On this ground, this work aimed at performing a comparative analysis between these two widely applied methodologies by generating both LB and SB models by using the same machine learning algorithm on a common dataset of 12789 hERG binders collected from the public repository ChEMBL (Mendez et al., 2019) and the commercial Excelra’s GOSTAR database (https://www.gostardb.com/). The training molecules were submitted to docking and rescoring calculations by using different docking engines. The resulting sets of scoring functions were used as input features for the SB model construction, while the LB model was trained on a set of physicochemical descriptors and molecular fingerprints. All the generated models were subjected to an internal evaluation by performing ten-fold cross-validation on the training set and validated on an external test set collecting hERG binders mined from literature.

## 2 Materials and methods

### 2.1 Dataset preparation

Data preparation and attributes calculations have been performed in a Konstanz Information Miner (KNIME) (Berthold et al., 2006) workflow published in our previous paper (Lunghini et al., 2022). Activity data were collected from the publicly available repository ChEMBL (Mendez et al., 2019) and the commercial Excelra’s GOSTAR database (https://www.gostardb.com/) (Zhao et al., 2020). The experimental activity data were retrieved from both databases by means of the ChEMBL identifier code “CHEMBL240.”

Only the measurements referred to “*homo sapiens*” or “*human*” have been retained. Censored values (i.e., > or <) have been excluded and only activity values reported as “IC50”, “EC50”, “Ki or “Kd” have been considered and converted into the negative log unit molar concentration (pK). Concerning the inhibition mechanism, some of the compounds contained in our dataset are competitive inhibitors. However, the information related to the mechanism of inhibition is not available for all the molecules. Therefore, we cannot exclude that our dataset includes also non-competitive or allosteric inhibitors.

Compound’s structures available as SMILES strings were preprocessed and standardized employing a Pipeline Pilot (v. 2018) protocol (BIOVIA, 2011, Dassault Systèmes, Pipeline Pilot version 2018, San Diego: Dassault Systèmes 2011., n. d.) following a standard chemical compounds cleaning procedure (Fourches et al., 2010), involving salts removal, standardization of functional groups and neutralization. Duplicates removal was carried out on the basis of the matching of standardized SMILES. When multiple activity measurements were available for a given compound, the median value was used as representative activity value (Parks et al., 2020).

The same protocol was applied for the preparation of the external test set collecting compounds retrieved from the study of Doddareddy et al. (2010). Specifically, only the compounds reporting the bioactivity data were retained and the molecules contained also in our training set (TS) and duplicates were removed resulting in 335 compounds.

By applying a threshold pK value of 5, the compounds were labeled as binders (pK ≥ 5) or non-binders (pK < 5) and the resulting compositions of the TS and the external test set are reported in Table 1. The pK threshold of 5, corresponding to the 10 μM, was chosen as this value is commonly employed in HTS campaigns to identify new hit compounds (Jahnke, 2007).

**TABLE 1:** Composition of the datasets used in this study.

Dataset	Binders	Non-binders
Training set	7303	5486
External validation set	231	89

### 2.2 Principal component analysis and scaffold analysis

To analyze the chemical space covered by our datasets, principal component analysis (PCA) was carried out by means of OriginPro 2022b (https://www.originlab.com/) on a set of 26 physicochemical descriptors as computed by the Vega ZZ suite v. 3.2.3 (Pedretti et al., 2021). The option “standardize scores” was checked and the first two components were plotted. Murcko scaffold analysis was performed by using DataWarrior v. 5.5.0 (Sander et al., 2015).

### 2.3 Ligand-based features calculation

The LB features were computed by using the RDKit node (https://www.rdkit.org/) available in KNIME (v. 4.5.2). In particular, 11 basic physicochemical properties (logP, TPSA, molecular weight, n° of rotatable bonds, n° of H-bond donors and acceptors, n° of heteroatoms, n° of atoms, n° of heavy atoms, n° of stereocenters and fraction of sp^3^ carbons) and ECFP6 and FCFP6 fingerprints, 1024 bits each, have been computed for each compound for a total of 2059 descriptors.

### 2.4 Docking simulations

All the docking simulations performed in this study were carried out by using the Cryo-EM structure of hERG channel in complex with astemizole (PDB ID 7CN1). The missing protein segments were modelled by using SWISS-MODEL webserver (Schwede, 2003), while missing atoms and hydrogens were added by means of VEGA suite of programs (Pedretti et al., 2021). The resulting protein structure was optimized by 10000 steps of energy minimization by keeping the backbone fixed, except for the modelled segments whose backbone was simulated unrestrained, to maintain the experimental folding. The so obtained structure was validated by i) generating the Ramachadran plot by means of PROCHECK v. 3.5 (Laskowski et al., 1993) and ii) estimating the overall quality factor as computed by ERRAT (Colovos and Yeates, 1993) (https://saves.mbi.ucla.edu/). Specifically, this parameter provides an estimation of the structure quality basing on non-bonded atomic interactions. The higher the score, the better the structure quality. The original structure was characterized by an overall quality factor of 78.375 (Figure S1A) and presented, according to the Ramachandran plot, 620 out of 822 residues in the favoured regions while 89 were found in the additional allowed regions (Figure S2). The overall quality factor increased to 83.789 in the optimized structure (Figure S1B). In this case, the Ramachandran plot (Figure S3) showed that 707 out of 1062 residues were in the favoured regions, while 199 and 20 residues were in the additional and generously allowed regions, respectively. Finally, six residues were located in the disallowed regions. Specifically, the residues found in the generously and disallowed regions belong to the modelled loops connecting the alpha helixes that form the pore and the voltage sensors of the channel, which lack a well-defined secondary structure.

Ligands structures were prepared by an automatic script in VEGA ZZ as described elsewhere (Beccari et al., 2017). All the possible stereoisomers were generated for the chiral molecules whose exact configuration was not specified. The so prepared molecules underwent docking calculations by means of three different programs: PLANTS v. 1.2 (Korb et al., 2006), LiGen v. 3.0 (Beccari et al., 2013) and GOLD v.5.8.1 (Jones et al., 1997).

In all the docking simulations, the binding site was set to contain the residues within 10 Å from the centre of mass defined by Tyr652, Thr623, Ser624 and Ser649, reported to be crucial for hERG activity (Mitcheson et al., 2008), and five poses were generated for each molecule. Re-docking calculations for pose validation were not performed as the structure of the ligand astemizole was not present in the employed structure due to its low EM density.

Concerning PLANTS docking, the calculation was performed by using the search speed1 and ChemPLP as fitness function. GOLD docking was carried out as reported elsewhere (De Luca et al., 2020; Vittorio et al., 2020) setting the virtual screening option as search efficiency and ChemPLP as scoring function. Finally, LiGen docking was executed employing CSopt as docking score for poses ranking as described in Manelfi et al. (2021).

### 2.5 Rescoring

All the poses computed by the three docking software were rescored by means of ReScore + tool (Pedretti et al., 2016) as implemented in VEGA ZZ, which allowed the calculation of 25 additional scoring functions including i) the different components of PLANTS and XScore scoring functions; ii) the MLP scores describing the hydrophobic contacts (Vistoli et al., 2010), iii) the Elect and ElectDD scores for the electrostatic interactions, iv) CVFF and CHARMM Lennard-Jones components for the evaluation of the van der Waals interactions and v) the Contacts scores which account for the number of the residues surrounding the bound ligand (Vistoli et al., 2017).

The scores computed by the docking and rescoring procedures were used to train RF models in two different ways. In the first case, for each score, only the value corresponding to the top ranked poses was considered for each compound. Concerning the stereoisomers, the scores values of the best poses of each isomer were averaged. Instead in the second case, we considered all the binding modes predicted by the three docking tools by computing for each scoring function the mean values over all the generated poses for each molecule. Regarding LiGen derived models, the pharmacophoric distances computed by the program were included in the model generation for a total of 55 descriptors.

### 2.6 Model building and validation

All the models described in this study were built by means of the Waikato Environment for Knowledge Analysis (WEKA v. 3.8.5) (Hall et al., 2009) by using Random Forest algorithm, employing the following settings: batch size = 100, numExecutionSlots = 1, maxDepth = 0 and numIterations = 100. Feature importance was assessed by mean impurity decrease approach.

Prior to the LB model generation, feature selection was carried out by applying the CfsSubsetEval algorithm in conjunction with the BestFirst search algorithm as implemented in WEKA. The CFSSubsetEval algorithm estimates the worth of a subset of attributes considering their individual predictive ability and the redundancy between features. Instead, the BestFirst algorithm searches attributes subset by exploiting a greedy search algorithm. The application of this features selection strategy resulted in 37 descriptors. Models combining LB and SB features were trained by employing the 37 selected LB attributes plus the SB features computed as described in the previous paragraphs.

All the generated models were subjected to an internal validation by performing ten-fold cross-validation on the entire training set and evaluated on an external dataset by using the following statistical parameters: Matthews Correlation Coefficient (MCC), accuracy (ACC), the area under curve of receiver operating curve (AUC), precision, sensitivity (SE) and specificity (SP). The equations employed for the calculation of such parameters are reported as follow:

$$MCC=\frac{TN\times TP-FN\times FP}{\sqrt{\left(TP+FP\right)\left(TP+FN\right)\left(TN+FP\right)\left(TN+FN\right)}}$$ (1)

$$ACC=\frac{TP+TN}{TP+FP+TN+FN}$$ (2)

$$Precision=\frac{TP}{TP+FP}$$ (3)

$$SE=\frac{TP}{TP+FN}$$ (4)

$$SP=\frac{TN}{TN+FP}$$ (5)

### 2.7 Applicability domain

The applicability domain was assessed by i) estimating the structural similarity and ii) by defining the interpolation space of the descriptors used to train the models. In the first case, the Tanimoto coefficient (Tc) was computed on a set of ECFP6 1024-bit fingerprints to estimate the similarity between training and test molecules. A given compound was considered inside the applicability domain if the Tc was higher than 0.7 (Ogura et al., 2019; Lunghini et al., 2022). Concerning the descriptors-based methods, we applied three different approaches included in the AMBIT software (http://ambit.sourceforge.net/index.html): range (Sahigara et al., 2012; García-Jacas et al., 2017), Euclidean distance (Klepsch et al., 2014; García-Jacas et al., 2017) and probability density (Tetko et al., 2006; Dimitriou-Christidis et al., 2008). A consensus approach was then used to define the applicability domain of the test molecules. In particular, a given compound was considered inside the applicability domain if it was inside the boundary of at least three methods.

## 3 Results

### 3.1 Analysis of the chemical space

**FIGURE 2:**
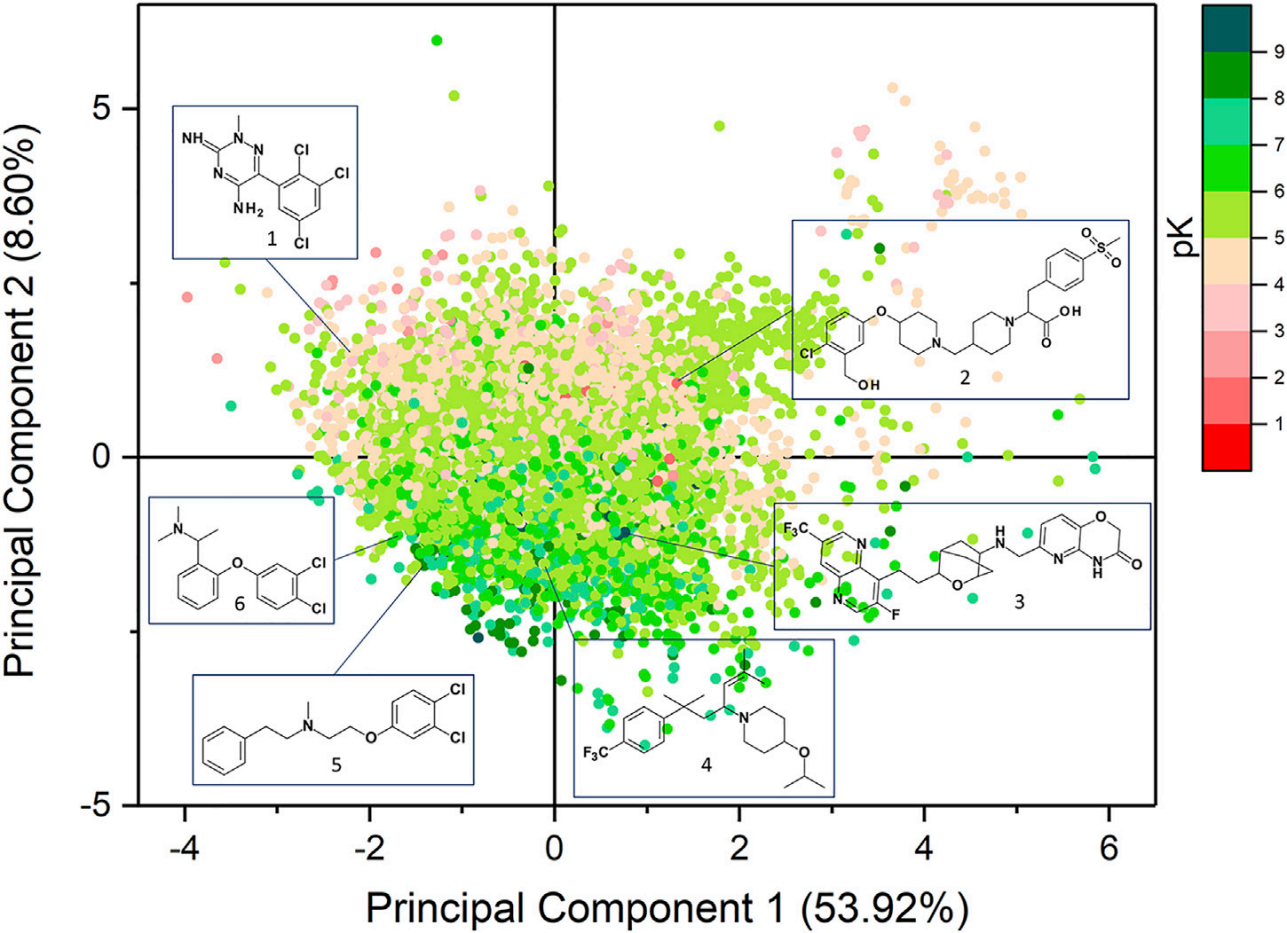
Scatter plots of the PCA analysis performed on the datasets used in this study.

Prior to models generation, PCA was performed to evaluate the diversity of the chemical space covered by the datasets employed in this study (Figure 2). The analysis was carried out using 26 physicochemical descriptors as computed by VEGA suite of programs. The cumulative variance expressed by the first two components was 62.52% (53.92% and 8.60%). The chemical space of the compounds of the training and external test sets is described by the PCA analysis reported in Supplementary Figure S4, which highlights that both datasets cover the same chemical space.

The first component is mainly driven by the molecular size as encoded by descriptors such as number of atoms, mass, number of bonds, surface and number of heavy atoms, which are all distributed along the *x*-axis. The polar surface area (PSA) and the number of H-bond donors (HBD) mostly influence the second component and they increase moving towards the upper *y*-axis. As expected, log P is also distributed on the PC2 but its values increase moving towards the negative *y*-axis. PSA and log P are the most influent properties which mostly contribute to the differentiation between binders and non-binders. Figure 2 highlights that the compounds with the highest affinity towards hERG (pK > 7) are mostly distributed in the lower part of the *y*-axis as they are more hydrophobic than the non-binders molecules. In Supplementary Table S2 the loadings values of the two principal components are reported.

Murcko scaffold analysis was carried out to identify the most frequent chemical scaffolds retrieved in hERG binders. As results, 6732 different Murcko scaffolds were identified and some of the most frequent frameworks are reported in Supplementary Table S3 with the relative average pK value and standard deviation (SD). Except for the phenyl ring which characterizes, with different substitution patterns, many common building blocks, the most frequent scaffolds carry at least one aromatic moiety and a tertiary or a secondary amine group that can be positively charged at physiological pH. This finding is in good agreement with several pharmacophore models described in literature (Cavalli et al., 2002; Aronov, 2006). The most recurrent framework retrieved in the high-affinity compounds consists of the 6-[[[4-[2-(1,5-naphthyridin-4-yl)ethyl]-3-oxabicyclo[2.2.2]octan-1-yl]amino]methyl]-4H-pyrido[3,2-b][1,4]oxazin-3-one that was found in 102 molecules of which 89 were hERG binders such as derivative **3** (Figure 2). Also, the N-(2-phenoxyethyl)-2-phenyl-ethanamine scaffold mainly characterizes binders as it was retrieved in 38 compounds among which 30 were binders like derivative **5** (Figure 2). Instead, the 4-phenoxy-1-[[1-(2-phenylethyl)-4-piperidyl]methyl]piperidine moiety was mostly detected in the non-binders molecules such as compound **2** (Figure 2). This framework appeared in 28 compounds of which 22 were non-binders. It is worth to note that derivative **2** carries a negatively charged carboxylic group which has been reported to reduce hERG affinity as it increases the polarity and is unable to form π-cationic interactions with the aromatic residues of hERG binding site (Wang et al., 2016). Another recurrent scaffold characterizing the non-binders was the 6-phenyl-2H-1,2,4-triazin-3-imine which was retrieved in 18 molecules and specifically in 15 low affinity compounds such as derivative **1** (Figure 2). As displayed by their mean pK values of ∼5 and SD lower than 0.6 (Supplementary Table S3), some scaffolds like the 1-(3-phenylpropyl)piperazine and the phenoxybenzene mainly characterized compounds with a medium affinity towards hERG, such as derivative **4** and **6**, respectively. The 1-(3-phenylpropyl)piperazine framework appeared in 25 compounds with a medium affinity and 25 non-binders, while the phenoxybenzene motif was detected in 60 molecules among which 56 are characterized by a medium affinity.

As shown in Supplementary Table S3, some frameworks are characterized by a SD value greater than 1 highlighting how the substitution patterns strongly influence the affinity towards hERG. Generally, increasing the polarity of the molecules leads to a detrimental effect on hERG binding as it hinders the capability of the molecule to interact with the hydrophobic residues of the pocket (Wang et al., 2016; Garrido et al., 2020).

### 3.2 Ligand-based classification models

In this study, a RF classification model was generated by employing a TS of 12789 compounds encoded by 2059 attributes including physicochemical descriptors and fingerprints. Before model generation, attributes selection was performed by using the CfsSubsetEval algorithm in conjunction with the BestFirst search algorithm as implemented in Weka software. Basing on the obtained results, 37 variables (Supplementary Figure S5) were selected comprising 7 physicochemical descriptors and 30 fingerprints that were subsequently exploited to train the model by means of Weka package using ten-fold cross-validation based on the training set to estimate the predictive performance of the model. As reported in Table 2, the obtained model displayed a good ability in discerning between binders and non-binders with a MCC value of 0.57, accuracy of 0.79 and AUC of 0.87. Specifically, the model was able to properly predict almost the 80% of the training molecules. Focusing on the misclassified compounds, we observed that these molecules were characterized by a low affinity towards hERG with a mean pK value of 5.10 and SD of 0.62. These compounds are characterized by physicochemical properties intermediate between those of the binders and non-binders, and this could justify why the model failed in correctly classifying them. The relevance of the selected descriptors for classification was analyzed and the outcomes are displayed in Supplementary Figure S5. As expected, logP is the most relevant attribute as hERG binders are characterized by lipophilic moieties, as described by many pharmacophores reported in literature (Cavalli et al., 2002; Ekins et al., 2002) which allow hydrophobic contacts at the binding site to be engaged. According to the results the fraction of Csp3 atoms is also relevant to discriminate between binders and non-binders. Most of hERG ligands carry aromatic rings that establish π-π interactions with the aromatic residues of the pocket thus being characterized by a low fraction of sp3 carbons (Garrido, et al., 2020). In this context, reducing aryl moiety and increasing the Csp3 fraction has been reported as strategy to decrease compounds affinity towards hERG (Cavalluzzi, et al., 2020). Features encoding for molecule polarity such as TPSA, H-bond donors and acceptors, were also identified as important for the classification. As described above, the increment of the polarity lead to an affinity loss, being therefore a crucial property to discern between hERG binders and non-binders. Overall, our results corroborated the SAR data available in literature concerning hERG ligands. Moreover, lipophilicity, TPSA, H-bond donors and acceptors were frequently retrieved as relevant features in other classification models (Raschi, et al., 2009; Yu, et al., 2016).

**TABLE 2:** MCC, Accuracy (ACC), AUC, Precision, Sensitivity (SE) and Specificity (SP) values of the generated classification models.

Evaluation metric	LB model	Best poses				Average scores			
		PLANTS	LiGen	GOLD	Consensus	PLANTS	LiGen	GOLD	Consensus
**MCC**	0.57	0.38	0.37	0.37	0.42	0.43	0.43	0.43	0.47
**ACC**	0.79	0.70	0.70	0.70	0.72	0.72	0.72	0.72	0.73
**AUC**	0.87	0.77	0.76	0.76	0.79	0.80	0.80	0.79	0.82
**Precision**	0.79	0.70	0.70	0.70	0.72	0.72	0.72	0.72	0.74
**SE**	0.84	0.82	0.83	0.82	0.85	0.82	0.83	0.83	0.85
**SP**	0.72	0.54	0.51	0.53	0.54	0.59	0.58	0.58	0.60

### 3.3 Structure-based classification models

The recent resolution of the experimental structure of hERG channel in complex with the inhibitor astemizole (PDB ID 7CN1) enables the development of SB predictive models for hERG-related cardiotoxicity which include information derived from the protein-ligand interactions. In light of this, the TS compounds were submitted to docking and rescoring procedures as described in the Methods section. The resulting scoring functions and the primary scores computed by each docking program were used to train RF classification models as described above for the LB classifier. Specifically, for each scoring function we considered i) only the score related to the top ranked docking pose and ii) the mean value computed over all the generated poses accordingly to the binding space concept (Vistoli et al., 2017). When LiGen was used, the 2.4% of TS molecules were not docked and therefore excluded from the models construction.

As displayed in Table 2, the models trained on the average scores (AV) showed better performances than those generated considering only the best poses (BP), which correspond to the highest scored pose, for each training molecule. Moreover, the classifiers developed from the results of the three docking tools displayed comparable performances with MCC values in the range of 0.37–0.38 for the BP models and of 0.43 for the AV models. In addition, we also generated consensus models by combining all docking and rescoring results, which led to a slight improvement of the performances for both BP and AV models with MCC values equal to 0.42 and 0.47, respectively. Specifically, we observed that the BP models are characterized by specificity values between 0.51 and 0.54 which highlighted the weakness of these classifiers in identifying non–binders. This parameter increases in the AV models and, in particular, in the consensus model which yielded a specificity value of 0.60. The low specificity values observed in these models might be attributed to the different distribution of the compounds in the two classes of the TS (Table 1) as the positive class contains more samples than the negative one. Overall, the rate of correct predictions, expressed by the accuracy values, is similar for all the docking-based models.

Taken together, the performances achieved by the SB classifiers are lower than those obtained by the model trained on the LB descriptors. Interestingly, the sensitivity observed in the docking-based models are comparable to those displayed by the LB classifier thus revealing a similar ability in classifying the binders class.

**FIGURE 3:**
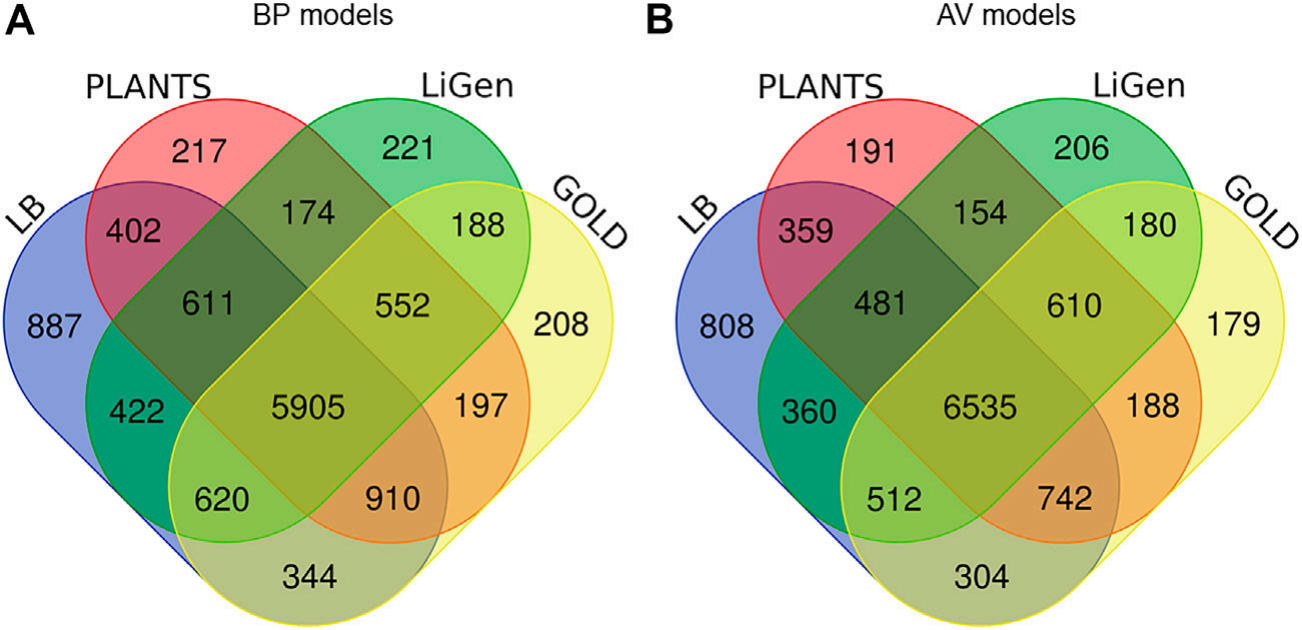
**(A)** Venn diagram displaying the number of TS molecules correctly classified by the LB classifier and **(A)** the BP SB models, **(B)** the AV SB models.

In Figure 3, the number of TS molecules correctly predicted by each model is reported. Concerning the BP models, 5905 compounds were correctly classified by both the LB and the docking-based models, while 887 molecules were rightly predicted only by the LB model (Figure 3A). Interestingly, some molecules are correctly predicted only by one of the SB classifiers. In more details, 217 molecules are correctly predicted only by PLANTS model, 221 by LiGen and 208 by GOLD. Moreover, there are pools of molecules correctly predicted by two of the SB models but not by the LB classifier. Specifically, 174 compounds were correctly classified only by LiGen and PLANTS models, 188 molecules were correctly predicted only by LiGen and GOLD while 197 compounds were correctly classified only by PLANTS and GOLD. Regarding the AV models, 6535 molecules were properly predicted by all the models, while 808 were rightly classified only by the LB model (Figure 3B). In this case 191 compounds were correctly predicted only by PLANTS-based model, 206 by LiGen and 179 by GOLD. In addition, 154 molecules were justly classified only by PLANTS and LiGen models, 180 only by LiGen and GOLD-based classifiers and 188 by GOLD and PLANTS models.

To rationalize the obtained results, the PCA analysis, performed as previously described, was mapped basing on the predictions obtained by the LB and BP models (Supplementary Figure S6). The obtained results highlighted that both LB and BP classifiers covered all the analysed chemical space without the formation of well-defined clusters of molecules correctly predicted only by a specific method.

**FIGURE 4:**
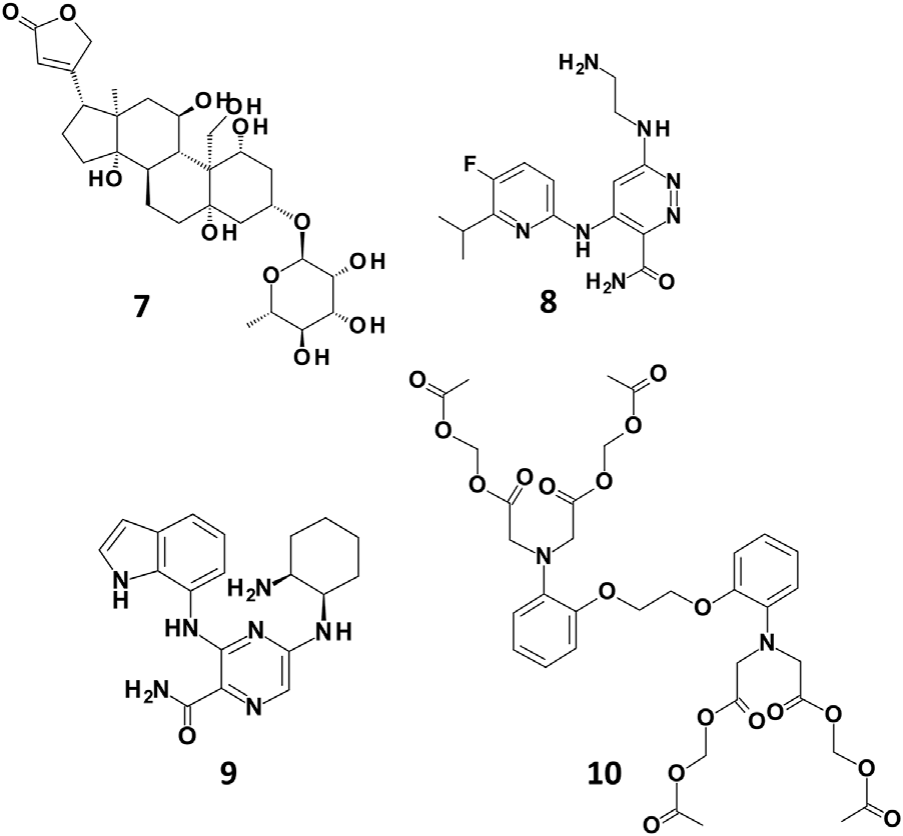
Chemical structures of some examples of compounds correctly predicted by one of the SB BP models (compounds **7**, **8** and **9**) and by all the three SB BP classifiers (compound **10**).

In order to better understand the different predictions observed for the docking-based models, we analysed the docking poses of some of the compounds rightly classified by only one of the SB BP models (compounds **7**, **8**, and **9** in Figure 4). For a comparison purpose, the binding mode of compound **10** (Figure 4) correctly predicted by all the three SB BP models was reported as well.

**FIGURE 5:**
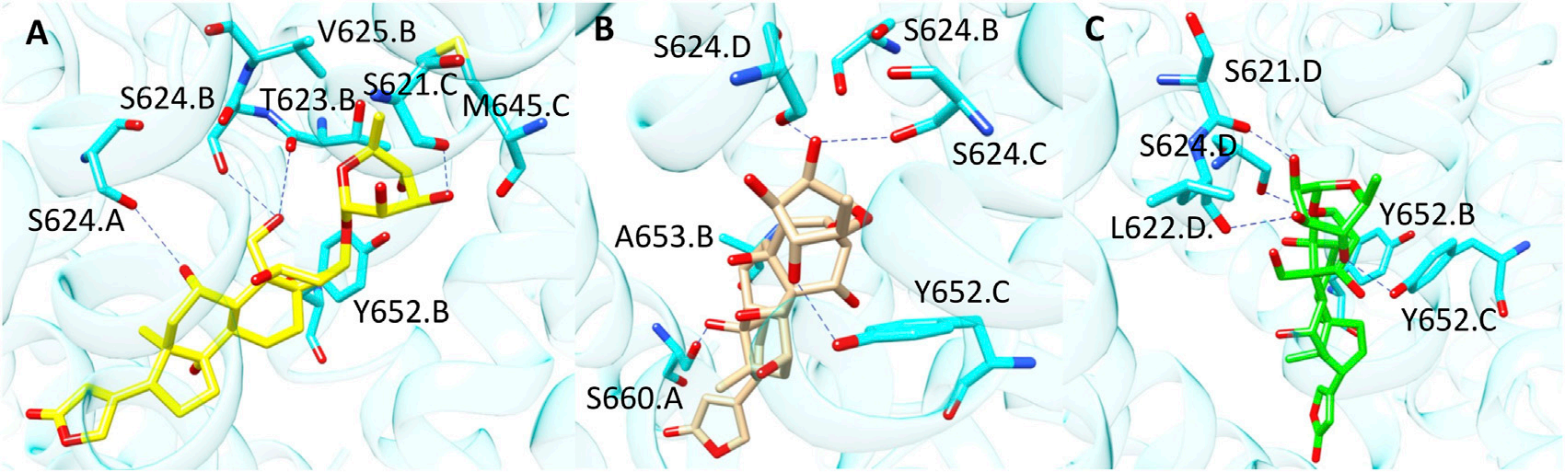
Binding modes of compound **7** into hERG binding site predicted by **(A)** PLANTS, **(B)** LiGen and **(C)** GOLD. H-bonds are represented as blue dashed lines.

Compound **7** (CHEMBL 222863) was correctly classified only by PLANTS derived model. This molecule belongs to the class of cardiac glycosides which are characterized by an aglycone steroid nucleus and a sugar moiety. It displayed a pK value of 5.24 and, differently from most of the hERG binders, is characterised by a negative logP value. Lipophilicity is an important parameter for drugs bioactivity as it affects the transport of the molecule through the membranes and the binding to their target. In a recent work, the influence of logP on the antiviral activity of cardiac glycosides was described highlighting that the percentage of active compounds was higher at ranges of logP values of −0.49–0.00 and 0.51–1.00. Furthermore, this study also pointed out that the length of the oligosaccharide chain, the nature and the configuration of the sugar moiety and the specific glycoside linkage are crucial for the biological activity of this class of compounds (de Pádua et al., 2022). Interestingly, compound **7** does not possess the typical chemical features of hERG binders such as a basic amine moiety and aromatic rings. Due to its physicochemical properties, the position of this derivative in the PCA plot is quite differentiated from the others compounds as displayed in Supplementary Figure S6. The analysis of the docking poses obtained from each docking tools showed that, according to PLANTS, the sugar moiety of **7** might be located in a region of the pocket lined by M645. C, S621.C, T623. B, V625.B and Y652.B, while the steroid portion is oriented towards Y652. A (Figure 5A). Instead, in LiGen pose, the sugar moiety is shifted towards S624.C and S624. D, while the steroid nucleus is situated close to S660. A and A653. B (Figure 5B). Finally, in the output yielded by GOLD compound **7** occupies hERG binding site with the sugar located in proximity of residues S621. D, L622. D and S624. D, while the steroid portion is close to Y652.B and Y652.C (Figure 5C). In all the three poses we could observe several H-bond interactions with residues that are reported to be crucial for hERG inhibition, such as T623, S624 and Y652 and hydrophobic contacts between the steroid nucleus and Y652. Furthermore, the binding mode provided by PLANTS enabled the formation of hydrophobic contacts between M645. C and V625.B and the methyl group of the sugar that are missed in the poses predicted by both LiGen and GOLD.

**FIGURE 6:**
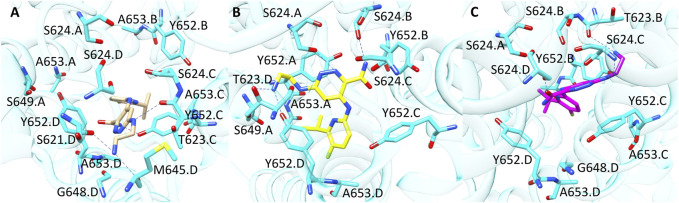
Binding modes of compound **8** into hERG binding site predicted by **(A)** LiGen, **(B)** PLANTS and **(C)** GOLD. H-bonds are represented as blue dashed lines.

Compound **8** (CHEMBL 3237581, pK 5.1) was correctly predicted only by LiGen and in the PCA plot is located close to a small cluster of molecules rightly predicted by the SB approach situated along the positive *y*-axes. According to LiGen outcomes, derivative **8** might occupy hERG binding site with the 5-fluoro-6-isopropyl–pyridine portion oriented towards S624.D, while the pyridazine ring is located close to Y652.C with the aminoethylamino chain inserted in the region lined by M645.D, G648.D, A653.D, S621.D and T623.C (Figure 6A). In the poses obtained from PLANTS, this region is occupied by the pyridine moiety while the rest of the molecules approaches Y652. A, S624. A and S624.B (Figure 6B). Instead, in the result generated by GOLD the pyridine ring is situated close to Y652.B while the pyridazine portion elicits π-π stacking with Y652.C (Figure 6C).

**FIGURE 7:**
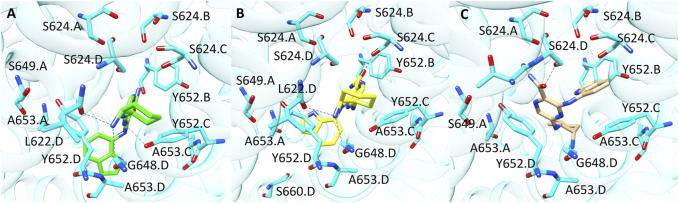
Binding modes of compound **9** into hERG binding site predicted by **(A)** GOLD, **(B)** PLANTS and **(C)** LiGen. H-bonds are represented as blue dashed lines.

Compound **9** (CHEMBL 3416024, pK 5.09) was rightly predicted only by GOLD-based model. Interestingly, the analysis of the docking poses revealed that the binding mode suggested by PLANTS and GOLD is quite similar as shown in Figure 7 (Panel A and B). In more details, the 2-aminociclohexyl portion is situated in proximity of L622. D and G648. D, while the pyrazine moiety contacts Y652. C. The main difference between the two binding modes relies on the arrangement assumed by the indole portion which is oriented towards A653. D in the pose obtained from GOLD and towards A653. A in the binding mode proposed by PLANTS. Despite this small difference, the pose yielded by GOLD provides more predictive scores. Instead, in the pose predicted by LiGen the indole moiety is shifted on the opposite side of the pocket towards Y652.B, while the pyrazine ring is close to Y652.D (Figure 7C). Finally, the cyclohexane is situated in a wide region of the pocket and does not contact any residue of hERG binding site.

**FIGURE 8:**
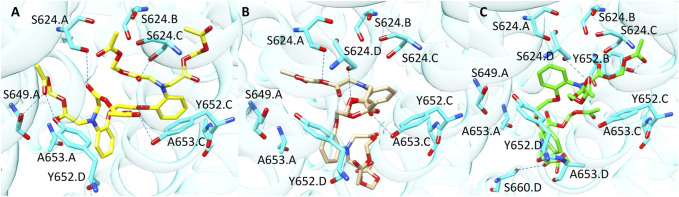
Binding modes of compound **10** into hERG binding site predicted by **(A)** PLANTS, **(B)** LiGen and **(C)** GOLD. H-bonds are represented as blue dashed lines.

Finally, compound **10** (CHEMBL 1608767, pK 5.88) was correctly classified by all the three SB BP models but not by the LB classifier. Among the molecules contained in our TS, compound **10** is characterised by the highest number of flexible torsions which is equal to 31. The results obtained from the three docking tools suggested that the presence of the four high flexible chains linked to the nitrogen atoms allows the simultaneous interaction with multiple sub-pockets of hERG binding site (Figure 8). Considering the high flexibility of compound **10**, this ligand might occupy hERG pocket adopting all the three conformations predicted by the different docking tools, thus furnishing reliable scores in all the three predictions and this could explain why this molecule was correctly predicted by all the three docking-based models.

Basing on the above-described results, we added the LB descriptors to the scoring functions computed during each docking and rescoring runs and trained the models on these new set of features by combining LB and SB descriptors. As displayed in Table 3, the addition of the LB features led to an improvement of the performances if compared to the classifiers trained on the sole scoring functions, which is mainly due to an enhancement of the specificity. Overall, the resulting models showed comparable performances and, similarly to the models built only on the scoring functions, the classifiers based on the average scores displayed a slightly better ability in discerning between binders and non-binders. Specifically, the best performing models were those built on the average scores of the poses computed by PLANTS and GOLD, which showed a MCC value of 0.54. However, the prediction performances of these new models remained still lower in respect to the LB predictor. In more details, the main difference relies on the capability of these classifiers to correctly identify the non-binders as expressed by the specificity value.

**TABLE 3:** MCC, Accuracy (ACC), AUC, Precision, Sensitivity (SE) and Specificity (SP) values of the classification models generated by combining the LB descriptors with the scoring functions calculated from each docking and rescoring calculation.

Evaluation metric	Best poses + LB descriptors				Average scores + LB descriptors			
	PLANTS	LiGen	GOLD	Consensus	PLANTS	LiGen	GOLD	Consensus
**MCC**	0.52	0.49	0.51	0.49	0.54	0.52	0.54	0.52
**ACC**	0.77	0.75	0.76	0.75	0.78	0.77	0.78	0.77
**AUC**	0.85	0.83	0.85	0.83	0.86	0.85	0.86	0.85
**Precision**	0.77	0.75	0.76	0.75	0.78	0.77	0.78	0.77
**SE**	0.84	0.84	0.84	0.85	0.84	0.85	0.84	0.85
**SP**	0.67	0.64	0.66	0.62	0.69	0.66	0.69	0.65

### 3.4 External validation

The ability of the generated RF classification models to discern between hERG binder and non-binders was further evaluated on an external test set containing 335 compounds tested on hERG channel and extracted from the dataset published by Doddareddy et al. (2010) as described under Materials and Methods section.

Considering that the performances achieved by the docking-based models were comparable, the docking calculations on the external test set were performed by using only LiGen. As results 320 molecules including 231 active and 89 inactive compounds were docked and submitted to rescoring calculations.

As shown in Table 4, in the external validation the best performance was achieved by the model trained on the average scores plus the LB descriptors which showed a MCC value of 0.22, ACC of 0.69 and AUC of 0.67. It is noteworthy that all the models involving the LB features performed better on the binders class as highlighted by the sensitivity which assumed values of 0.78–0.79, while they failed in recognizing the non-binders as revealed by the specificity values lower than 0.5. Conversely, the classifier trained on the average scores, which displayed a MCC of 0.21, as obtained for the LB model, performed better on the non-binders class as suggested by its specificity value of 0.70, while its capability to predict the binders is comparable to a random classification as highlighted by its sensitivity value of 0.53. Instead, a similar ability in predicting the two classes was denoted for the BP model which displayed a specificity value of 0.60 and sensitivity of 0.61 on the external dataset.

**TABLE 4:** Evaluation of the performances of the classifiers on the external test set.

Evaluation metric	LB	BP		AV	
		LiGen	LiGen + LB	LiGen	LiGen + LB
**MCC**	0.21	0.19	0.15	0.21	0.22
**ACC**	0.68	0.60	0.67	0.58	0.69
**AUC**	0.61	0.62	0.66	0.63	0.67
**Precision**	0.68	0.68	0.66	0.69	0.69
**SE**	0.78	0.61	0.79	0.53	0.79
**SP**	0.43	0.60	0.35	0.70	0.43

Remarkably, the model based on average scores plus LB descriptors showed performances slightly better than the LB model, a result never seen when analysing the training set. This result emphasizes the role of docking-based descriptors when screening external molecules which are not seen during the model training.

Following a standard machine learning procedure, we also split our starting dataset into 70% for training and the remaining 30% for testing. Specifically, the test set compounds were chosen by using the Diversity Picker node of RDKit as implemented in KNIME to select molecules diverse from those used in the learning phase. We trained the RF models on the new TS and validated them i) by ten-fold cross-validation, ii) on the new generated test set and iii) on the external validation set from Doddareddy et al. As shown in Table S4, the results yielded by the cross-validation were comparable to those obtained by training the models on the entire dataset, highlighting the robustness of our ML classifiers. The outcomes of the validation of the new generated models on the new test set showed the same trend of that obtained from the cross-validation, with the LB model showing the best performance over all the developed new models. Interestingly, the evaluation on the external test set from Doddareddy et al., revealed again that the model combining the average scores and the LB-features displayed the best performance with a MCC value of 0.28 (Supplementary Table S5). Moreover, the results pointed out that the performance of the LB model decreased respect to that observed for the model trained on the entire dataset (Table 4), with a reduction of the MCC value from 0.21 to 0.07 (Supplementary Table S5). Instead, the LiGen AV-based models returned similar performances in terms of MCC to those reported in Table 4, despite an increase of the sensitivity and a decrease of the specificity were observed. Overall, the resulting outcomes revealed that the best performing classifiers were those built on the entire dataset and, therefore, applicability domain evaluation was performed only for these models as reported in the next section.

### 3.5 Applicability domain and comparison with other classifiers

**FIGURE 9:**
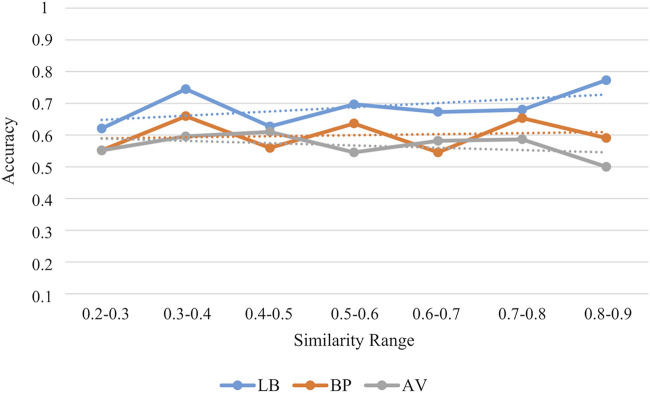
Accuracy values for the external test set in each similarity range. Similarity is estimated in terms of Tanimoto score.

The applicability domain of our classification models was evaluated by using a consensus approach of four different methods: i) structural similarity (Ogura et al., 2019; Lunghini et al., 2022), ii) range (Sahigara et al., 2012; García-Jacas et al., 2017) iii) Euclidean distance (Klepsch et al., 2014; García-Jacas et al., 2017) and iv) probability density (Tetko et al., 2006; Dimitriou-Christidis et al., 2008). While the first method relies on the compounds structure, the others approaches are based on the descriptors used to train the models. Specifically, a molecule was considered to be inside the applicability domain if it fulfilled at least three methods. In Table 5, the compounds inside the applicability domain according to each strategy and the consensus approach are reported. Basing on these results, the SB models displayed a wider applicability domain if compared to the models built on the LB features. Furthermore, we analysed the relationship between the accuracy of the prediction and the similarity of the molecules contained in the external test set in respect to the training compounds. As results, a positive trend was observed for the LB model as the prediction accuracy rises as the similarity between training and test molecules increases (Figure 9). This positive trend was not detected for the SB models thus further highlighting that their predictive performance does not rely on the structural similarity with the molecules used in the training phase.

**TABLE 5:** Number of compounds of the external validation set inside the applicability domain according to each strategy and the consensus approach.

Model	Similarity	PCA range	Euclidean distance	Probability density	Consensus
**LB**	97	187	320	276	217
**LiGen BP**	97	296	318	304	298
**LiGen BP-LB**	97	153	320	243	183
**LiGen AV**	97	291	319	299	292
**LiGen AV-LB**	97	181	320	275	216

**FIGURE 10:**
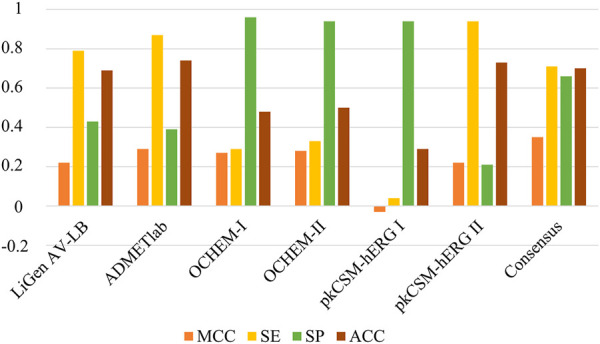
Comparison of the performances between LiGen AV-LB model and other tools available in literature (ADMETlab, OCHEM and pkCSM).

Finally, the performance of the LiGen AV-LB classifier was compared with other hERG models available in literature: ADMETlab (https://admetmesh.scbdd.com/), OCHEM (https://ochem.eu/) and pkCSM (https://biosig.lab.uq.edu.au/pkcsm/). We focused on these classifiers since they do not include the Doddareddy’s dataset in their training set. As shown in Figure 10, when applied to our test set, the analysed models provided MCC values comparable to our model LiGen AV-LB. In more details, ADMETlab, OCHEM-I and OCHEM-II yielded MCC values of 0.29, 0.27 and 0.28, respectively. Instead, a negative MCC of −0.03 was obtained applying pkCSM-hERG I model, while pkCSM-hERG II furnished a MCC value of 0.22 like LiGen AV-LB. Moreover, we observed that LiGen AV-LB, ADMETlab and pkCSM-hERG II showed higher sensitivity and accuracy values. Conversely, OCHEM models and pkCSM-hERG I displayed higher specificity values. These outcomes pointed out that each model is able to properly classify only one of the two classes. Based on these results, we decided to apply a consensus strategy by combining the predictions obtained with the different models. Specifically, a given compound was predicted to be a hERG binder, if at least three models classified it as binder. As shown in Figure 10, the consensus approach returned the best performance as expressed by the MCC value of 0.35 as well as more balanced sensitivity and specificity values of 0.71 and 0.66, respectively. Overall, these outcomes highlighted the beneficial effect of considering multiple approaches to predict hERG liability.

## 4 Discussion

In this work, we exploited both LB and SB strategies to develop RF machine learning models for hERG-related cardiotoxicity of drugs. To this aim, we employed a comprehensive dataset composed of 12789 hERG binders including 6732 Murcko scaffolds, thus covering a large chemical space. The SB classifiers were built on a set of scores describing the different protein-ligand interactions as computed by docking and rescoring procedures involving three diverse docking tools: PLANTS, LiGen and GOLD. In a standard docking approach, the best pose is usually selected to perform the analysis; however, it is widely recognized that often the docking tools fail in correctly score and ranking the generated binding poses, and that a ligand might assume multiple binding states within the binding site. Several studies highlighted that considering different binding modes represents a winning strategy to improve the predictive ability of docking scores (Vistoli et al., 2017; Mazzolari et al., 2020). Therefore, to account for ligand mobility within hERG pocket, for each score we computed the average value over all the predicted binding poses generated by each docking tool. The results gained from the ten-fold cross-validation performed on the TS emphasized that the three employed docking tools yielded models with comparable performances. Notably, the predictive ability of the models increased when the average scores were employed in the learning step confirming the beneficial effect of considering multiple binding orientations. Nevertheless, the best performance was achieved by the LB classifier. However, PCA analysis displayed that both LB and SB approaches are able to cover all the analysed chemical space. Moreover, compounds like **7**, that lack the classical features of hERG binders, was correctly predicted by PLANTS model, while the LB approach fails in correctly classified it. Another interesting case is represented by compound **10** which is, with 31 flexible torsions, the most flexible molecule of the TS, due to the presence of four flexible chains connected to the two nitrogen atoms. This compound was correctly classified by all the docking-based models but not by the LB classifier. The analysis of the binding poses provided by each docking software highlighted that hERG inhibitors might elicit several H-bonds mainly with S624, Y652 and T623. Moreover, Y652 could also be involved in hydrophobic and aromatic interactions with the lipophilic and aromatic moieties characterizing hERG binders. It was not surprisingly that the compounds misclassified by all the approaches showed a pK average value of 4.95 with a SD of 0.53, being therefore characterized by a low affinity towards hERG channel. As displayed in Figure 1, molecules with these pK values share similar properties and can be easily misclassified by the machine learning models. To improve this aspect, future studies are addressed to investigate the use of different activity thresholds that might lead to a better discrimination between binders and non-binders.

Differently from the cross-validation outcomes, the application of our models on an external dataset revealed that the best performance was achieved by the model combining the LB features with the average scores. Considering that cross-validation is performed on the same set of molecules used to build the model, it is easy understandable that the LB model, which is based on the physicochemical properties of the compounds used in the training phase, provides a better performance if compared to a SB model. Instead, when applied to unseen molecules, our study demonstrated that the integration of both approaches can enhance the performances of both LB and SB models. Although multiple cross-validation is considered as the standard strategy to assess the predictive power of a RF model, this study suggests that such a strategy can introduce biases when comparing LB and SB models.

Some aspects might be considered concerning the docking-based classifiers. The structure employed for the docking studies has a low resolution of 3.70 Å and, therefore, do not furnish a good atomic model of the protein. Secondly, induced-fit effects were not considered in our study as the protein was kept rigid in our docking calculations. Therefore, the obtainment of an experimental structure of hERG channel with higher resolution and the use of a set of diverse hERG conformations might contribute to increase the predictive performance of docking scores.

Applicability domain evaluation based on four different methods, including structural similarity, range, Euclidean distance and probability density, highlighted that the SB models are characterized by a wider applicability domain respect to the LB-based classifiers.

Finally, the comparison with other hERG models publicly available, ADMETlab, OCHEM-I and II, and pkCSM-hERG I and II, pointed out that all the models showed comparable performances in terms of MCC values, except for pkCSM-hERGI which yielded a negative MCC. Furthermore, the outcomes revealed that each model is able to better recognize only one of the two classes, as expressed by the unbalanced specificity and sensitivity values. Instead, the application of a consensus strategy based on the combination of the different predictions yielded by the different methods, led to more balanced specificity and sensitivity which resulted in an improvement of the overall performance. Therefore, the use of a consensus approach represents a valuable strategy to prioritize both sensitivity and specificity, thus allowing to obtain better predictions respect to the use of a single model. Our models were made available to the scientific community at the following Zenodo repository: https://doi.org/10.5281/zenodo.7551782.

## 5 Conclusion

In this work, several binary RF classification models for hERG liability were developed by using both LB and SB approaches and the combination thereof. To the best of our knowledge, our study is the first work describing a straightforward comparison between LB and SB machine learning models for hERG-related cardiotoxicity trained on the same dataset including 12789 hERG binders. The TS molecules were submitted to docking and rescoring procedure which provided a set of scoring functions that were exploited to build the SB classifiers. The ten-fold cross-validation on the TS pointed out that the all the SB models were characterized by comparable performances and, specifically, the models trained on the average scores outperformed those built considering only the best docking pose. Moreover, the combination of LB and SB attributes led to an improvement of the models performances. However, the LB model proved to be the best predictive model in the cross-validation. PCA analysis highlighted that both approaches covered all the analysed chemical space, even if some molecules were correctly predicted by only the LB or one or all of the SB models. Interestingly, both the LB and the LiGen AV model showed the same MCC of 0.21 on the external dataset, while a slight improvement of the performance was achieved by the model combining the LB and average scores which displayed a MCC value of 0.22. Furthermore, a positive trend was observed between the accuracy values of the LB classifier and the structural similarity between training and test molecules conversely to the docking-based models, thus emphasizing that the predictive ability of the SB models does not rely on the similarity with the TS compounds. Overall, our results suggested that the integration of docking scores and LB descriptors can improve the performance of both LB and SB classifiers when applied to unseen molecules. Finally, this study emphasized that the validation by external datasets is of crucial relevance to obtain unbiased performance evaluations when comparing LB and SB approaches.

## Data Availability

The models and the datasets containing the descriptors used to train the models presented in this study can be found in online repositories: https://doi.org/10.5281/zenodo.7551782.
