# Supplementary material for: Ensemble of structure and ligand-based classification models for hERG liability profiling
Source: Front Pharmacol. 2023 Mar 23;14:1148670. doi: 10.3389/fphar.2023.1148670 (PMC10076575; doi:10.3389/fphar.2023.1148670)
Supplement: Supplementary file 1 [file DataSheet1.PDF]

Ensemble of structure and ligand-based classification models for hERG liability profiling

Serena Vittorio[1], Filippo Lunghini[2], Alessandro Pedretti[1], Giulio Vistoli[1], Andrea R. Beccari[2*]

[1]Dipartimento di Scienze Farmaceutiche, Università degli Studi di Milano, Via Mangiagalli, 25, I-20133 Mila-no, Italy

[2]EXSCALATE, Dompé Farmaceutici SpA, Via Tommaso de Amicis 95, 80123 Napoli, Italy

*** Correspondence:**
Corresponding Author
andrea.beccari@dompe.com

Table of contents

Table S1 Key information of some of the computational models for hERG liability profiling reported in literature........ 2

Figure S1. ERRAT plots........ 3

Figure S2. Ramachandran plot of the template structure........ 4

Figure S3. Ramachandran plot of the optimized structure........ 5

Figure S4. PCA analysis of the chemical space of the compounds of training and external test set........ 6

Table S2. Loadings of descriptors from PCA analysis........ 7

Table S3. Most frequent Murcko frameworks retrieved in the datasets employed in this study........ 8

Figure S5. Features selected to train the LB models and their relative importance........ 9

Figure S6. PCA analysis mapped basing on the predictions from the different models........10

Table S4. Performances of the classification models obtained after 70:30 splitting of the entire dataset into training and test set........ 11

Table S5. Evaluation of the classification models obtained after 70:30 splitting of the entire dataset on the external validation set from Doddareddy *et al.* 11

Table S1 Key information of some of the computational models for hERG liability profiling reported in literature.

Authors	Type	Descriptors	Dataset size		Validation metrics			Reference
			Training	Test	R^2	R	ACC	
**Ekins *et al.***	Pharmacophore	-	15	22	0.83*	-	-	10.1124/jpet.301.2.427
**Cavalli *et al.***	Pharmacophore	-	31	6	0.74*	-	-	10.1021/jm0208875
**Zhang *et al.***	ML	CDK fingerprints and molecular descriptors	927	236	-	-	0.8475*	10.1039/c5tx00294j
**Lee *et al.***	ML	Physicochemical descriptors and ECFP4 fingerprints	2130	10	-	-	0.8*	10.1186/s12859-019-2814-5
**Zhang *et al.***	ML	Morgan and MACCS fingerprints	10850	2570	-	-	0.840*	10.1021/acs.jcim.2c00256
**Du-Cuny *et al.***	ML	Physicochemical descriptors	93	54	0.59*	-	-	10.1021/ci200271d
**Arab *et al.***	ML	2D descriptors	8380	499	0.67*	-	-	10.35248/2157-7463.22.13.006
**Creanza *et al.***	ML	GLIDE and GOLD docking scores and protein-ligand interaction fingerprints	8337	100	-	-	0.79*	10.1021/acs.jcim.1c00744
**Meng *et al.***	ML	Interaction features, ECFP2, MACCS and EState fingerprints	9215	496<br>403<br>203<br>44	-	0.765**	-	10.1016/j.tox.2021.153018

*validation performed on the test set

**result from cross-validation

A Overall quality factor**: 78.375

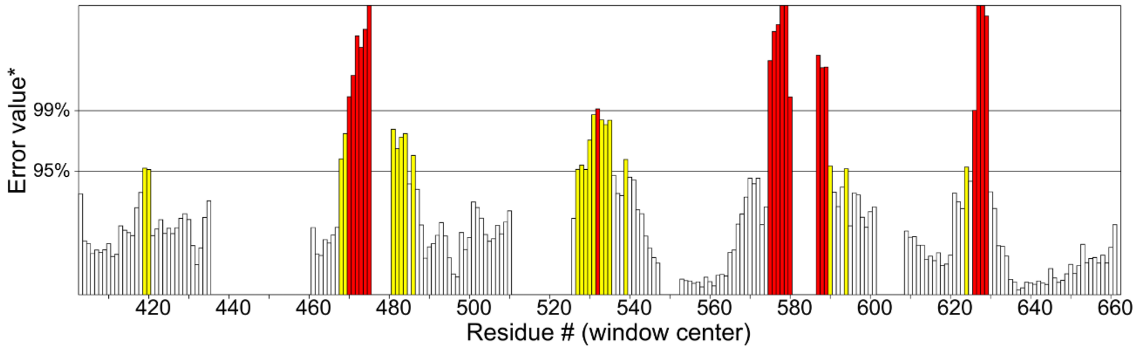

*On the error axis, two lines are drawn to indicate the confidence with which it is possible to reject regions that exceed that error value.

**Expressed as the percentage of the protein for which the calculated error value falls below the 95% rejection limit. Good high resolution structures generally produce values around 95% or higher. For lower resolutions (2.5 to 3A) the average overall quality factor is around 91%.

B Overall quality factor**: 83.789

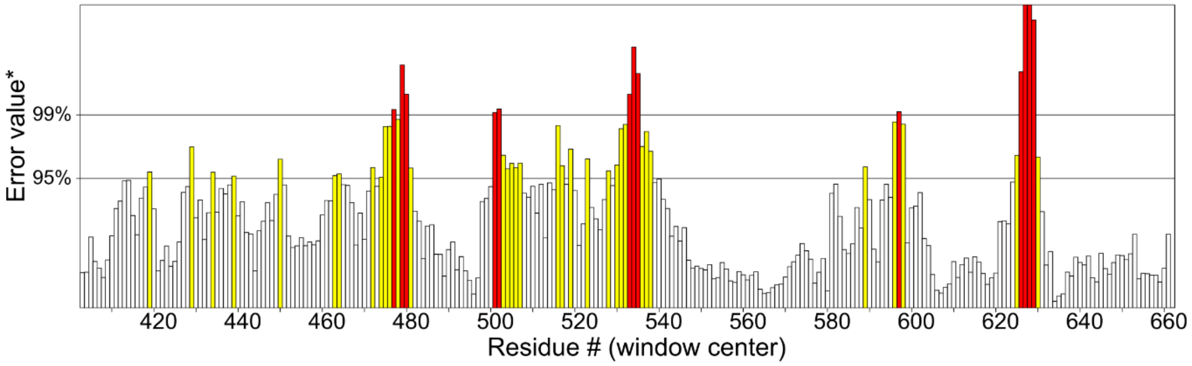

*On the error axis, two lines are drawn to indicate the confidence with which it is possible to reject regions that exceed that error value.

**Expressed as the percentage of the protein for which the calculated error value falls below the 95% rejection limit. Good high resolution structures generally produce values around 95% or higher. For lower resolutions (2.5 to 3A) the average overall quality factor is around 91%.

Figure S1. ERRAT plots of the template structure, PDB ID 7CN1 (Panel A), and the optimized structure (Panel B). The protein residues are reported in the y-axis, while the error value is indicated in the y-axis. An error value higher than 99% imply low quality regions.

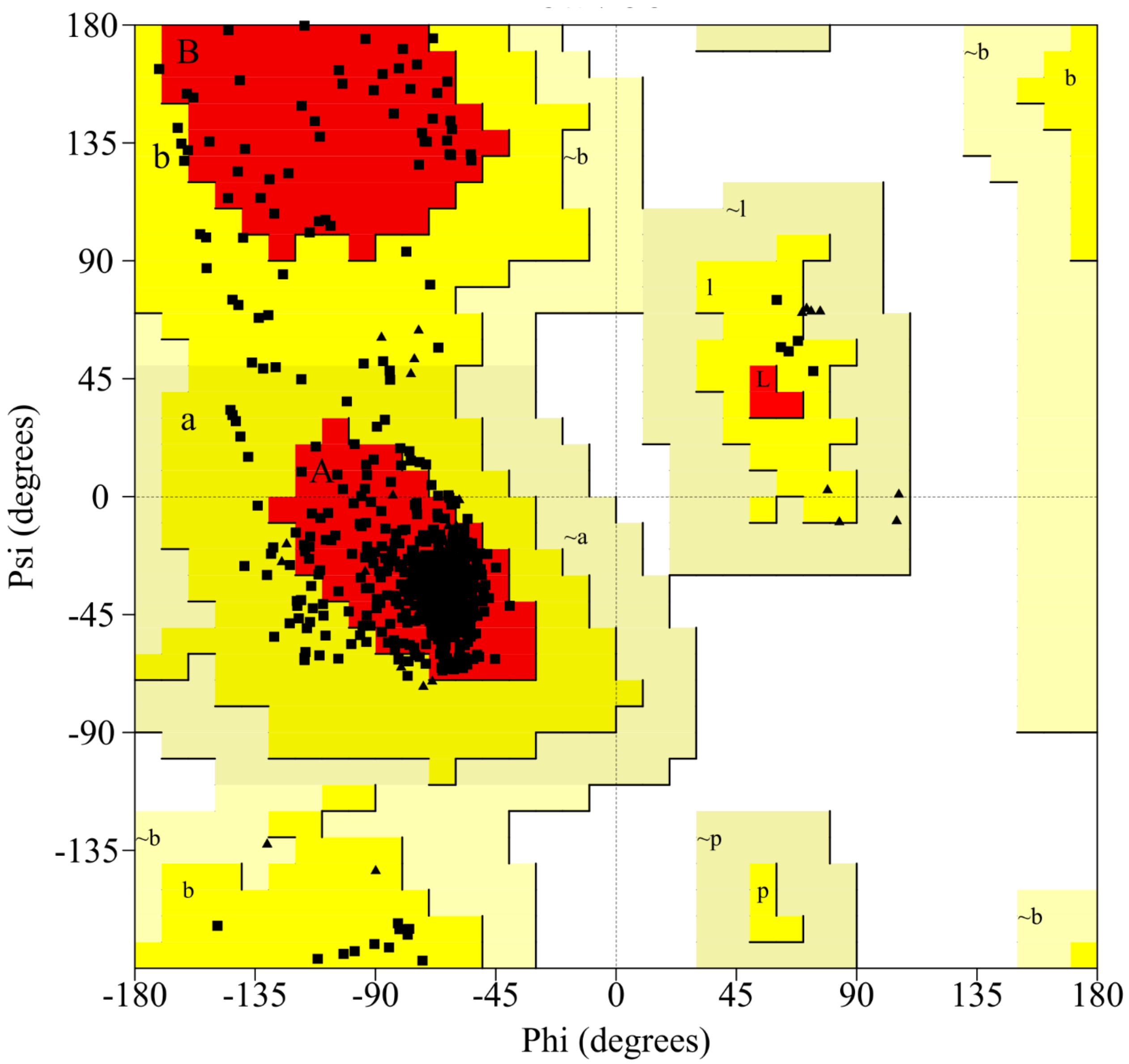

Plot statistics

Residues in most favoured regions [A,B,L]	620	87.4%
Residues in additional allowed regions [a,b,l,p]	89	12.6%
Residues in generously allowed regions [~a,~b,~l,~p]	0	0.0%
Residues in disallowed regions	0	0.0%
	----	------
Number of non-glycine and non-proline residues	709	100.0%
Number of end-residues (excl. Gly and Pro)	45	
Number of glycine residues (shown as triangles)	43	
Number of proline residues	25	

Total number of residues	822	

Figure S2. Ramachandran plot of the template structure (PDB ID 7CN1).

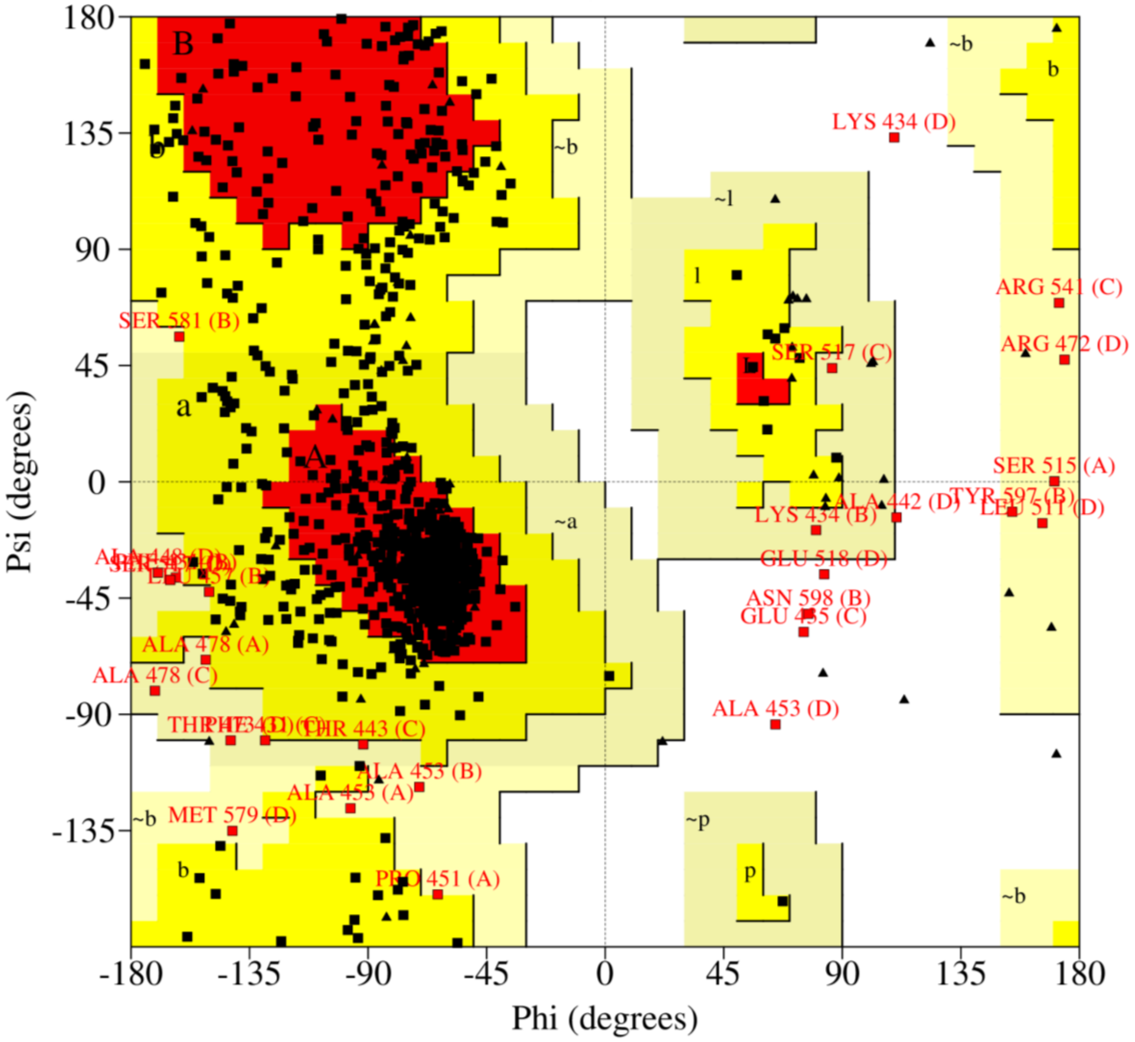

Plot statistics

Residues in most favoured regions [A,B,L]	707	75.9%
Residues in additional allowed regions [a,b,l,p]	199	21.4%
Residues in generously allowed regions [~a,~b,~l,~p]	20	2.1%
Residues in disallowed regions	6	0.6%
	----	------
Number of non-glycine and non-proline residues	932	100.0%
Number of end-residues (excl. Gly and Pro)	10	
Number of glycine residues (shown as triangles)	76	
Number of proline residues	44	

Total number of residues	1062	

Figure S3. Ramachandran plot of the optimized structure.

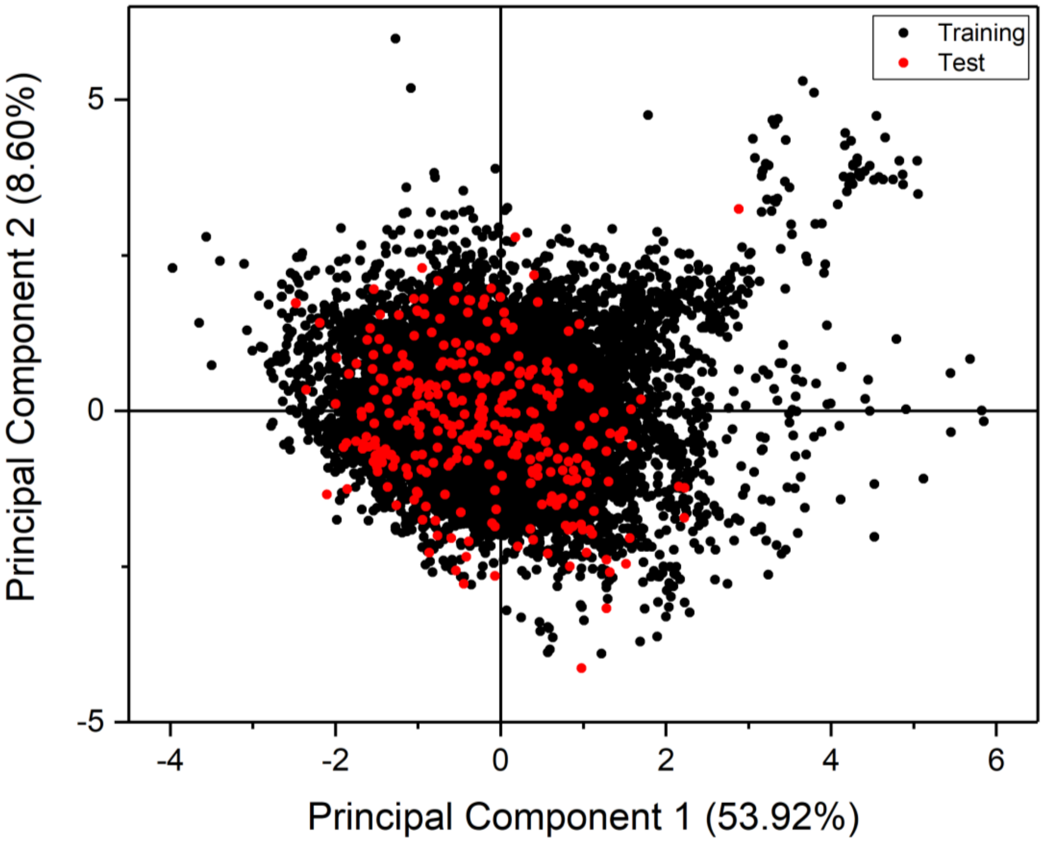

Figure S4. PCA analysis of the chemical space of the compounds of training and external test set.

Table S2. Loadings of descriptors from PCA analysis.

Descriptor	PC1	PC2
Angles	0.24501	-0.00345
Atoms	0.25255	-0.00609
Bonds	0.25407	-0.01554
Charge	0.06923	0.12598
Chiral atoms	0.10064	0.22750
Dipole	0.08430	0.08199
EZ Bonds	-0.00635	0.04242
FlexTorsions	0.18037	-0.00580
Gyrrad	0.17727	-0.14464
HbAcc	0.15462	0.25422
HbDon	0.06755	0.44957
HeavyAtoms	0.25422	-0.02971
Impropers	0.09593	0.30327
Lipole	-0.04497	-0.05117
Mass	0.24968	-0.01614
Ovality	0.25256	-0.0421
PSA	0.14835	0.42936
Rings	0.14892	-0.12293
SAS	0.25304	-0.0810
SAV	0.25988	-0.05235
Sdiam	0.25911	-0.04457
Surface	0.25912	-0.02906
Torsions	0.21727	-0.09814
Vdiam	0.25881	-0.05219
LogP	0.06070	-0.55769
Volume	0.25933	-0.03086

Table S3. Most frequent Murcko frameworks retrieved in the datasets employed in this study.

Murcko Scaffold	Frequency	pK mean
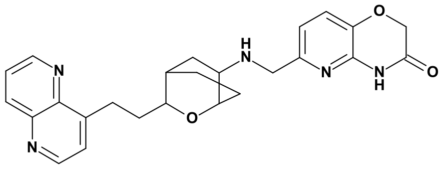	102	6.56±1.52
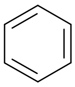	78	5.46±1.29
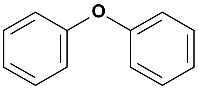	60	5.56±0.37
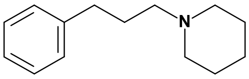	50	5.14±0.56
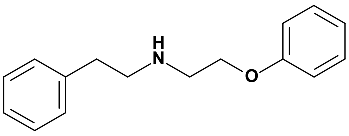	38	6.62±1.71
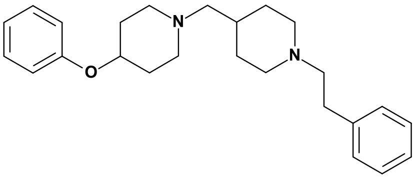	28	4,41±0.87
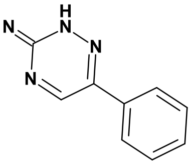	18	4.66±0.40

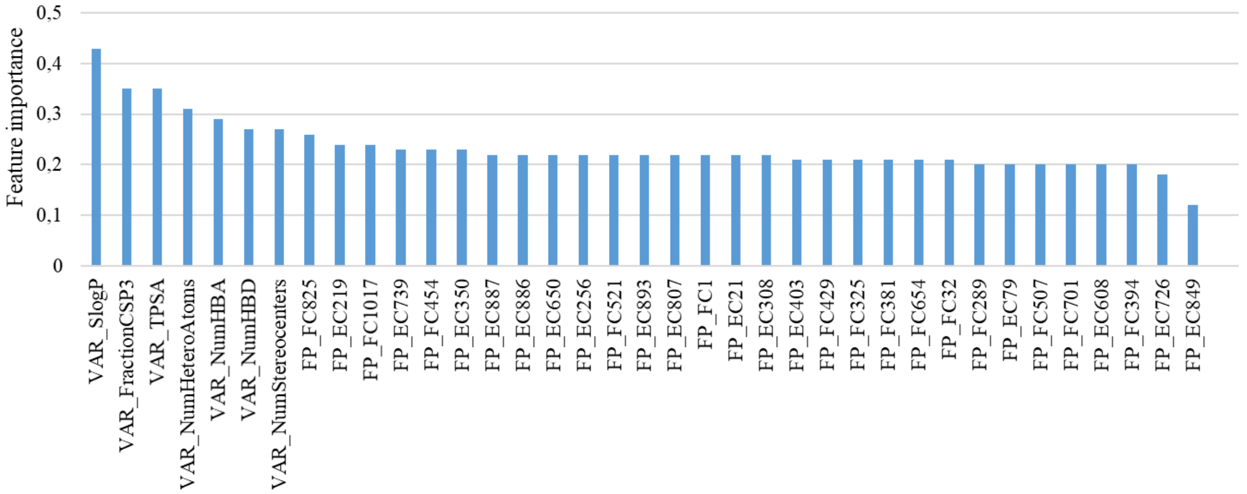

Figure S5. Features selected to train the LB models and their relative importance.

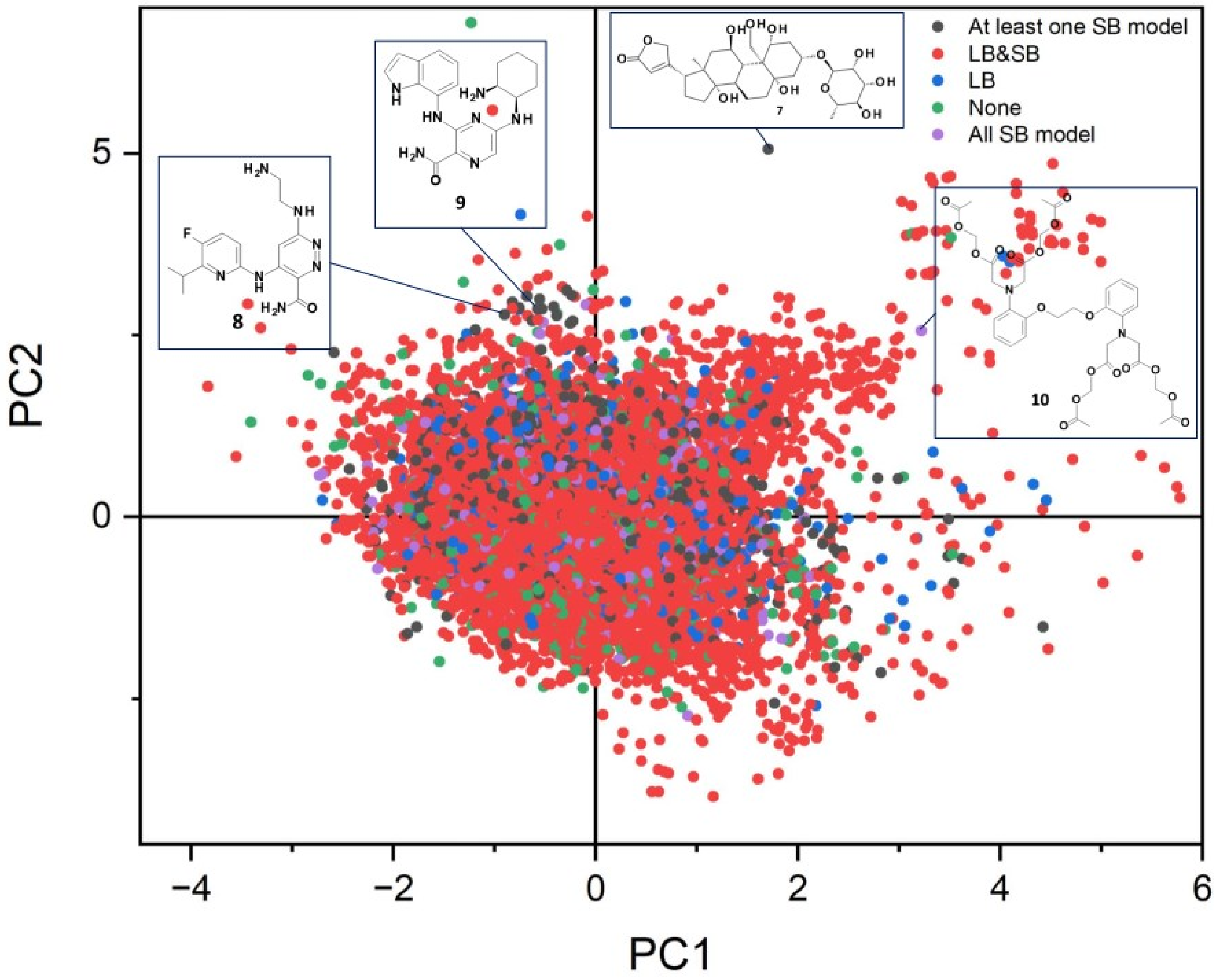

Figure S6. PCA analysis mapped basing on the predictions from the different models. The analysis concerns the predictions made by the LB and SB BP models. Points colours as coded as follow. Black points indicate compounds correctly classified by at least one of the SB models, while red points represent molecules correctly classified by the LB and at least one docking-based models. Compounds rightly predicted by only the LB model and by all the three BP SB model are represented in blue and purple, respectively, Molecules misclassified by all the approaches are coloured in green.

Table S4. Performances of the classification models obtained after 70:30 splitting of the entire dataset into training and test set.

Evaluation metric	10 fold cross-validation					Test set				
	LB	LiGen BP	LiGen BP+LB	LiGen AV	LiGen AV+LB	LB	LiGen BP	LiGen BP+LB	LiGen AV	LiGen AV+LB
**MCC**	0.61	0.40	0.52	0.46	0.55	0.42	0.29	0.36	0.32	0.40
**ACC**	0.81	0.71	0.77	0.74	0.79	0.71	0.65	0.69	0.67	0.70
**AUC**	0.89	0.78	0.85	0.81	0.87	0.79	0.70	0.77	0.73	0.78
**Precision**	0.81	0.71	0.77	0.74	0.79	0.72	0.65	0.69	0.67	0.71
**SE**	0.87	0.86	0.87	0.87	0.88	0.81	0.82	0.81	0.80	0.82
**SP**	0.73	0.50	0.63	0.56	0.66	0.60	0.44	0.53	0.51	0.56

Table S5. Evaluation of the classification models obtained after 70:30 splitting of the entire dataset on the external validation set from Doddareddy *et al.*

Evaluation metric	LB	LiGen BP	LiGen BP+LB	LiGen AV	LiGen AV+LB
**MCC**	0.07	0.10	0.14	0.21	0.28
**ACC**	0.66	0.60	0.70	0.65	0.74
**AUC**	0.62	0.63	0.68	0.65	0.69
**Precision**	0.63	0.64	0.66	0.69	0.71
**SE**	0.83	0.65	0.89	0.70	0.88
**SP**	0.24	0.46	0.21	0.53	0.36
